# 

**Published:** 1860-08

**Authors:** 


### DENTAL REGISTER

### OF THE WEST.

EDITED BY

J. TAFT AND GEO. WATT.

August, 1860.

MONTHLY.--THREE DOLLARS PER ANNUM IN ADVANCE.

CINCINNATI:

J. T. TOLAND, PUBLISHER AND PROPRIETOR.

J. TAFT, DENTIST, No. 56 WEST FOURTH STREET.

